# Evaluation of Microcrystalline Cellulose Derived from *Saccharum officinarum* L. (Sugarcane) Leaves as a Disintegrant in Tablet Formulations

**DOI:** 10.34172/apb.2020.050

**Published:** 2020-05-11

**Authors:** Julie Ann S. Ng

**Affiliations:** College of Pharmacy, Riverside College, Inc., BS Aquino Dr, Bacolod, 6100 Negros Occidental, the Philippines.

**Keywords:** Disintegrant, FTIR, Microcrystalline cellulose National formulary, Organoleptic, pH, Sugarcane

## Abstract

***Purpose:*** Complete recycling of the crop residues of sugarcane in the Philippines remains to be achieved. This study purposed to derive microcrystalline cellulose (MCC) from sugarcane leaves and test its disintegrating properties in tablet formulation.

***Methods:*** *Saccharum officinarum* L. (sugarcane) leaves were used to prepare MCC powder. According to the conventional method, the preparation of cellulose powder requires heating the raw material with acid and alkali followed by washing, bleaching, and sieving. Hydrolysis of the bleached product was carried out using hydrochloric acid to obtain MCC powder, and the physicochemical properties of the produced MCC powder were studied including its organoleptic properties, pH value, %loss on drying, %water soluble substances and Fouriertransform infrared (FTIR) spectrum.

***Results:*** The resulting powder was evaluated for its disintegrating property in the preparation of blank tablets, which were compared to tablets prepared using commercially available MCC. MCC powder derived from sugarcane leaves had properties at par with commercially available MCC and was in conformance with National Formulary (NF) specifications.

***Conclusion:*** Disintegrating properties were also significantly better than the commercially available MCC.

## Introduction

Sugarcane (*Saccharum officinarum* L. ) is a well-known crop in the family Poaceae. It is a perennial grass that commonly found in tropical South Asia and Southeast Asia (indigeneous). It has a thick longitudinal stalk, which is generally 3 to 5 m in height, approximately 5 cm in diameter, and is characterized by its sweet taste due to its high sucrose content.^1^

Sugarcane is mostly grown in subtropical and tropical regions around the world includes the both sides of the equator, to approximately 35° N and 35° S In 2007, the main sugarcane-producing countries were Brazil (33% of the world’s production), India (23%), China (7%), Thailand (4%), Pakistan (4%), Mexico (3%), Colombia (3%), Australia (2%), the United States (2%) and the Philippines (2%). The area of sugarcane cultivation is globally rising in response to increasing demands for bioethanol and sugar demand for consumption.^2^

Sugarcane grows for 12 to 16 months before being harvested between June and December each year. Sugarcane harvesting methods include both green and burnt cane harvest. In some cane-growing areas, it is not impossible to harvest cane green. The leftover cuttings form mulch, which keeps in moisture, stops the growth of weeds and prevents the soil erosion. Meanwhile in other areas, the sugarcane will be burnt to remove leaves, weeds and other matter.^3^

Sugarcane field burning is the most common harvesting method because it makes the process easier and requires less manual labor. In the burning process, the field is set to fire to and the leaves are burned off the stalks. About 80% of the “trash,” including straw, the tops, and green and dry leaves, is burned off. These components constitute about 25% of the entire sugar cane stalk. The burning kills microorganisms and burns the trash, both of which keep the soil rich when left in the fields.^4^

Over the years, sugarcane field burning has made smoke emissions from mechanical harvesting an issue. The burning of biomass becomes the major source of toxic gases. It generates several products during the process and cause adverse effects on the health of all the living populations. The products can be particulate matter, carbon monoxide, polycyclic aromatic hydrocarbons (PAHs), organic acids, aldehydes, ozone and inorganic chemical species, volatile and semi-volatile compounds of nitrogen and sulphur. Due to the production of several toxic compounds and it effects on causing health problems during the process of sugar cane burning, it is very important to focus on fine and ultra-fine particulate matters (PM10 and PM2.5). These particulate matters consist of a mixture of liquids, gases, and solids deposited on particles, i.e. PAHs. These matters can be derived from the incomplete process of organic combustion, and can cause mutagenic and carcinogenic effects.^5^

In the Philippines, sugarcane leaves were considered agricultural waste and burned in the field. To best eliminate the problem associated with the burning of this agricultural waste, the problem must be turned into a resource. The research undertaken focuses on the derivation of microcrystalline cellulose (MCC) from sugarcane leaves and testing the cellulose’s disintegrating property when used in a tablet formulation.

## Materials and Methods

### Extraction of cellulose

A total of 500 g of the sugarcane leaves were treated with 10 L of nitric acid. The resulting material was filtered and immersed in sodium hydroxide in a controlled water bath set at 100°C for 3 hours. The resulting material was further digested with 8 L of sodium hydroxide solution for 1 hour at 80°C. This was thoroughly washed with distilled water and filtered. The product was then bleached with 10 L of sodium hypochlorite for 2 hours at 40^o^C. The bleached sample was thoroughly washed with distilled water until it was neutral to litmus paper. It was filtered and then dried in the oven at 60^o^C for 16 hours. The product was sifted through a no. 40 sieve, dried further at 60°C for 1 hour, after which it was stored in a closed container.^6^

### Production of MCC

A 226 g quantity of α-cellulose collected was placed in an Erlenmeyer flask and hydrolyzed with 4.5 L of hydrochloric acid, at a boiling temperature of 105°C for 15 minutes. The mixture of hot acid was transfered into 13.5 L of cold tap water. It was then followed by vigorous stirring using a stirring rod and incubated overnight. The MCC obtained through this process was then filtered and washed with water until it became neutral. After that, it was filtered, pressed, and dried in the oven at a temperature of 60°C for 16 hours.^7^

### Physicochemical properties of the microcrystalline cellulose derived from sugarcane (MCC-SC)

#### Organoleptic characteristic

The organoleptic characteristic (odor and color) were examined in accordance with NF specifications.

#### Identification test

Iodinated zinc chloride solution was prepared by dissolving 20 g of zinc chloride and 6.5 g of potassium iodide in 10.5 mL of water. Next, 0.5 g of iodine was added and shaken for 15 minutes. Then, 10 mg of MCC was placed on a watch glass and dispersed in 2 mL of the iodinated zinc chloride solution.^8^

#### pH determination

A total of 1 g of the powder material was shaken with 50 mL of distilled water for 5 minutes. Then, the pH of the liquid supernatant was measured using a pH meter.^8^

#### Loss on drying

A total of 3 g of the powder sample was transferred into the evaporating dish. After that, it was dried in the oven at 105^o^C until a constant weight. The percentage of moisture content was determined as the ratio of the weight of moisture loss to the weight of the sample.^8^

#### Water soluble substances

A total of 5 g of the sample was shaken with approximately 80 mL of water for 10 minutes and was filtered through Whatman No. 42 filter paper into a tared beaker. The residue was washed with 20 mL of water and was evaporated to dryness on a steam bath. Dry at 105°C for 1 hour, cooled, weighed and calculated as a percentage.^8^

#### Fourier-Transform Infrared (FTIR) absorption

A sample of MCC-SC was ground in the mortar for reducing the average of the particle size to 1 or 2 microns. Then, total of 5 to 10 mg of finely ground sample was placed onto the face of a KBr plate. After that, a small drop of mineral oil was added in and the second window was placed on the top. It was then rubbed with a gentle circular, back‐and‐forth rubbing motion of the two windows, the mixture was then distributed between the plates. The sandwiched plates were placed in the spectrometer and a spectrum was obtained. The KBr plates were thoroughly cleaned after the first sample. The windows were wiped with a tissue and then washed several times with methylene chloride followed with ethanol. The procedure was repeated again for Comprecel^®^ M102 (D50), the commercially available MCC.^9^ The result between the extracted MCC and that of the commercially available MCC was compared.

#### The purity of MCC-SC

#### Presence of sugar

To 10 mg of the sample, 2 mL of Benedict’s reagent was placed in the test tube. It was then heated in the boiling water bath for 3-5 minutes. The procedure was repeated for Comprecel^®^ M102 (D50).^10^ The result between the extracted MCC and that of the commercially available MCC was compared.

#### Presence of starch

To 10 mg of the sample, 2 drops of 0.1N iodine VS was added. The procedure was repeated again for Comprecel^®^ M102 (D50).^10^ The result between the extracted MCC and that of the commercially available MCC was compared.

#### Preparation of granules

Granules were prepared by wet granulation with acacia as a binder, with lactose as diluent. The targeted weight of each tablet was 170 mg. Commercially available MCC and MCC-SC were used as disintegrants for comparative study.^11^

#### Preparation of tablets

The granules were compressed into a tablet using an automated tableting machine (MANESTY model F) at a compression force of 50kN. After ejection, the tablets were stored over silica gel for 24 hours to allow the elastic recovery and hardening.^11^

#### Evaluation of tablets

#### Weight test

The weight of each individual tablet was determined by dusting each tablet off with a camel-hair brush and placing it in the analytical balance. This procedure was repeated for twenty tablets in triplicates. The data from the tablets were analyzed for the sample mean and Relative Standard Deviation (RSD).^12^

#### Hardness test

Twenty blank tablets were selected randomly from each batch for this test. PHARMATEST PTB 311E 3 in 1 Hardness, Diameter and Thickness Tester was used. The test was done in triplicates. The mean value and RSD as a measure of variation were calculated.^13^

#### Friability test

A total of 6.5 g of blank tablets were weighed together in an analyticalbalance. These tablets were placedin PHARMATEST PTF 1DR friabilator and rotatedfor 4 minutes at 25 rpm. The percentage loss in weight was calculated for each batch. The test was performed over triplicates.^14^

#### Disintegration test 148

Six tablets were randomly selected from each of the batches and place in each of the tubes in the basket of PHARMATEST PTZ AUTO 2 Two Position Semi-Automated disintegration unit. The time taken for the tablet to break down into particles and pass through the mesh was noted. The mean disintegration time was calculated.^15^

## Results and Discussion

### Physicochemical properties of MCC-SC

The organoleptic characteristics, identification test result, pH, loss on drying (%) and water-soluble substances (%) has complied with the NF specifications for MCC are as shown in Table 1.

**Table 1:** Physicochemical properties of MCC-SC

	**Acceptance Criteria**	**MCC-SC**
Organoleptic characteristic odor	Odorless	Odorless
Color	White	White
Identification test	Turns violet-blue with iodinated zinc chloride	Turns violet-blue with iodinated zinc chloride
pH determination	Between 5.0 and 7.5	6.63±0.01
Loss on drying	Loses NMT 7.0%	6%
Water soluble substances	NMT 0.24%	0.18%±0.01

### FTIR absorption

**Figure 1:**
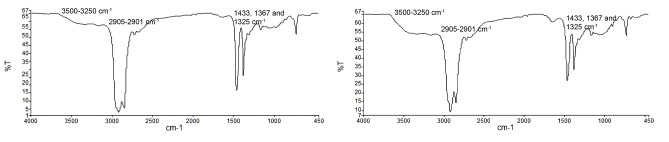
Spectrum graph of Comprecel® (left) and MCC-SC (right).

The spectrum graph of commercially available MCC and MCC-SC in Figure 1 showed similarities between both band intensity and position. The the spectra analysis with reference to published data showed several typical features of MCC. An absorption band at 3500-3250 cm^-1^ indicated the characteristic intermolecular and intramolecular OH stretching vibration band in the spectra. The presence of peak from 2905-2901 cm^-1^ showed the presence of more crystalline order in both MCCs. The peak at 1433, 1367 and 1325 cm^-1^ was associated with the intermolecular hydrogen bonds at the C group and at the OH in the plane bending vibration.^16^

The spectrum graph of MCC-SC indicates that the sample was successfully derived from the raw plant origin in comparison to commercially available MCC preparation of different plant origin (Figure 1).

### Purity of MCC-SC

In the test for the presence of sugar, the color of Benedict’s reagent was not change and there was no formation of brick red precipitate indicating that sugar was not detectable in commercially available MCC and MCC-SC samples.

In the test for the presence of starch, there was no appearance of purplish to blue color after the addition of Iodine TS.^17^ This indicates that starch was not detectable in commercially available the MCC and MCC-SC samples.

The negative results to the sugar and starch tests as shown in Table 2 indicate that MCC in the commercially available form and that derived from sugarcane leaves have no sugar or starch impurities.

**Table 2:** The purity of commercially available MCC and MCC-SC

	**Commercially available MCC**	**MCC-SC**
Sugar	Not detectable	Not detectable
Starch	Not detectable	Not detectable

### Evaluation of tablets

The data for tablet weight in Table 3 showed significant differences (*P* < 0.05) among each other. The tablets with a formulation containing 5% commercially available MCC, 3% and 5% MCC-SC were found to comply with the official compendial specifications for uniformity of tablet weight (170.00±7.5%).^18^ The tablets with a formulation containing 7% MCC-SC had substandard weight. Weight is a function of the quality of granulation, the flow of granules and machine performance.^18^ Out of the three factors, only the flow of granules differed among the formulations. Generally, the principles of looking into the true densities of the various MCC grades are similar. Small particles that are more heterogeneous in size could achieve closer packing.^19^ The close packing of particles results in particles being stacked on top of one another causing problem in the formation of the tablet during compression. To enhance the flow of granules with different concentration of MCC per formulations, 1% magnesium stearate was added as a lubricant to each formulation. Magnesium stearate as a lubricant can be added in 1 to 5%. But in this case, only the minimum amount was added because when MCC is mixed with magnesium stearate, the tablet strength can be weakened significantly as the amount of added lubricant increase. Similar results are also observed with other lubricants.^20^ The results for tablet weight indicate that utilizing the lesser concentration of 3% and 5% MCC-SC provides a better result than the 7% MCC-SC.

**Table 3:** Tablet weight

	**Weight (mg)**	**SD**	***P*** **value**
Comprecel® 5%	171.68	5.47	0.000
MCC-SC 3%	174.88	2.57	0.000
MCC-SC 5%	165.69	3.74	0.000
MCC-SC 7%	148.72	3.67	0.000

3%, 5%, and 7% indicate the formulation that contain a commercially MCC (Comprecel®) or MCC-SC as disintegrant; n=20 in three replicates

The data for tablet hardness, friability and disintegration time in Table 4 showed that tablet hardness was highest with the formulation containing 5% commercially available MCC. The hardness exhibited by the tablet formulated was within USP 39 specifications (4-10 kgf). It also possessed acceptable resistance according to USP 39 specifications (<1%) to abrasion, chipping and breakage. However, it took the longest time to disintegrate as compared to other formulations. A formulation containing 3%, 5% and 7% MCC-SC produced a tablet with hardness, friability and disintegration time complying with the official compendial specifications. The hardness, however, decreased along with the raising of the disintegrant concentration. Generally, as there is an increase in weight variation, there is also a decrease in hardness and an increase in friability.^21^ Thus, a formulation containing a lower concentration of 3% and 5% MCC-SC produced a tablet with higher resistance to abrasion, chipping and breakage than the tablets with 7% MCC-SC that also resulted to substandard weight tablet. Formulations with MCC-SC also exhibited better disintegrating property than the formulation with commercially available MCC at 5%. The results showed that the MCC-SC is cost-effective with consideration given that sugarcane leaves are considered trash^4^ and with the acquired results from this study showing that the MCC-SC extracted using the conventional way conformed to NF specification and displayed better disintegrating property at lower concentration compared to the commercially available MCC.

**Table 4:** Tablet hardness, friability & disintegration time

	**Hardness ( kgf )**	**SD**	***P*** **value**	**Friability (%)**	**SD**	***P*** **value**	**Disintegration time (min)**	**SD**	***P*** **value**
Comprecel® 5%	9.97	0.54	0.000	0.51	0.01	0.000	18.11	2.52	0.000
MCC-SC 3%	8.01	0.56	0.000	0.45	0.005	0.000	15.40	3.29	0.000
MCC-SC 5%	6.80	0.44	0.000	0.55	0.01	0.000	13.45	2.47	0.000
MCC-SC 7%	4.82	0.50	0.000	0.74	0.01	0.000	6.07	1.71	0.000

3%, 5%, and 7% indicate the formulation that contain a commercially MCC (Comprecel®) or MCC-SC as disintegrant

## Conclusion

The study successfully produced MCC from the α-cellulose extracted from sugarcane leaves. MCC powder derived from sugarcane leaves meets the National Formulary (NF) standard. Moreover, there are no impurities as to sugar and starch content. There is also no remarkable difference to the commercially available MCC of different plant origin. The tablet formulation containing commercially available MCC used as a disintegrant at 5% had higher value for hardness hence, the formulation that used MCC-SC at 3%, 5%, and 7% had a shorter disintegration time and worked better as a disintegrant to the commercially available MCC.

## Ethical Issues

Not applicable.

## Conflict of Interest

None declared.

## Acknowledgments

The author wishes to thank Mr. Virgilio Y. Tan II for his unwavering support and insight throughout this study. To Riverside College Inc. and the University of the Philippines Manila, without whom the access to the laboratory, instruments, and equipment this research would not have been possible. And finally, the author wishes to thank all close friends and family for their encouragement and support.

## References

[1] Singh A, Lal UR, Mukhtar HM, Singh PS, Shah G, Dhawan RK (2015). Phytochemical profile of sugarcane and its potential health aspects. Pharmacogn Rev.

[2] Cheavegatti-Gianotto A, de Abreu HM, Arruda P, Bespalhok Filho JC, Burnquist WL, Creste S (2011). Sugarcane (Saccharum X officinarum): a reference study for the regulation of genetically modified cultivars in Brazil. Trop Plant Biol.

[3] Australia: Bundanberg Sugar, https://www.bundysugar.com.au/education/process/harvesting.html. 2019. Accessed 21 March 2019.

[4] Brittany C, Christopher C, Max K, Viet N. Sugarcane. Pre-harvest Burning. https://sites.google.com/site/sugarcanepm/pre-harvest-burning. 2009. Accessed 21 March 2019.

[5] Silveira HC, Schmidt-Carrijo M, Seidel EH, Scapulatempo-Neto C, Longatto-Filho A, Carvalho AL (2013). Emissions generated by sugarcane burning promote genotoxicity in rural workers: a case study in Barretos, Brazil. Environ Health.

[6] Akhabue CE, Osubor NT (2017). Optimization of extraction of microcrystalline cellulose from orange peel waste using response surface methodology. Ife J Sci.

[7] Ohwoavworhua FO, Adelakun TA (2010). Non-wood fibre production of microcrystalline cellulose from Sorghum caudatum: characterisation and tableting properties. Indian J Pharm Sci.

[8] U.S. Pharmacopoeia-National Formulary [USP 39 NF 24]. Volume 4. Microcrystalline cellulose. Rockville, MD: United States Pharmacopeial Convention, Inc; 2015. p. 7231.

[9] Nuance. How to prepare IR sample? http://www.nuance.northwestern.edu/docs/keckii-pdf/how-to-prepare-ir-samples.pdf. 2019. Accessed 21 March 2019.

[10] Keshk SMAS, Haija MA (2011). A new method for producing microcrystalline cellulose from Gluconacetobacter xylinus and kenaf. Carbohydr Polym.

[11] Setu NI, Mia Y, Lubna NJ, Chowdhury AA (2014). Preparation of microcrystalline cellulose from cotton and its evaluation as direct compressible excipient in the formulation of Naproxen tablets. Dhaka Univ J Pharm Sci.

[12] U.S. Pharmacopoeia-National Formulary [USP 39 NF 24]. Volume 1. Weight variation. Rockville, MD: United States Pharmacopeial Convention, Inc; 2015. p. 2051.

[13] U.S. Pharmacopoeia-National Formulary [USP 39 NF 24]. Volume 1. Tablet breaking force. Rockville, MD: United States Pharmacopeial Convention, Inc; 2015. p. 1610.

[14] U.S. Pharmacopoeia-National Formulary [USP 39 NF 24]. Volume 1. Tablet friability. Rockville, MD: United States Pharmacopeial Convention, Inc, 2008. p. 1609.

[15] U.S. Pharmacopoeia-National Formulary [USP 39 NF 24]. Volume 1. Disintegration. Rockville, MD: United States Pharmacopeial Convention, Inc; 2015. p. 537.

[16] Haque SM, Chowdhury AA, Rana AA, Masum SM, Ferdous T, Rashid MA (2015). Synthesis of microcrystalline cellulose from pretreated cotton obtained from Bombax ceiba L and its characterization. Bangladesh J Sci Ind Res.

[17] FAO. Microcrystalline cellulose. http://www.fao.org/3/w6355e/w6355e0l.htm. 2019. Accessed 21 March 2019.

[18] Ozioko C. Quality Control Test for Tablets. https://www.pharmapproach.com/quality-control-tests-for-tablets/. 2019. Accessed 21 March 2019.

[19] Soh JL, Yang L, Liew CV, Cui FD, Heng PW (2008). Importance of small pores in microcrystalline cellulose for controlling water distribution during extrusion-spheronization. AAPS PharmSciTech.

[20] Morin G, Briens L (2013). The effect of lubricants on powder flowability for pharmaceutical application. AAPS PharmSciTech.

[21] Rajani C, Kumar DD, Jaya D, Kumar JA (2017). Effects of granule particle size and lubricant concentration on tablet hardness containing large concentration of polymers. Braz J Pharm Sci.

